# Taming the Chinese Dragon: A Promising Cornerstone for Transatlantic Trade Cooperation?

**DOI:** 10.1007/s10272-021-0944-2

**Published:** 2021-01-26

**Authors:** Simon J. Evenett

**Affiliations:** MBA — Intern. Trade and Economic Developm., University of St. Gallen, Blumenbergplatz 9, 9000 St. Gallen, Switzerland

## Abstract

The desire of many policymakers to tame the Chinese Dragon is apparent. But what is particularly disappointing is the little, if any, reflection on how to induce the government in Beijing to change course, whether with respect to domestic policy or foreign economic policy.

Even by the standards of politicians, President Trump pursued his international policy goals in a transactional, self-centred and unilateralist manner. His administration represented a clear break with the past: no longer would the United States support multilateralism, nor would it necessarily honour security guarantees, nor would it respect global and regional trade rules. Even if previous administrations had, from time to time, been selective in their support of the liberal world economic order and for democracy, there has never been an American administration that has conducted itself in the manner witnessed over the past four years.

European nations, both inside and out of the European Union, have not escaped President Trump's wrath. European steel producing nations were among those slapped with import tariffs on the unsubstantiated grounds that their exports constituted a threat to the national security of the US. Car exporters were threatened with similar treatment but for some reason were ultimately spared. Beyond the trade policy sphere, the Trump administration's contempt for the European Union and NATO was palpable.

Only in the second half of President Trump's four-year term did US officials begin seriously exploring joint approaches with European counterparts to so-called level playing field concerns in the world trading system. Largely, these concerns were taken to involve various forms of subsidies received by Chinese firms. In parallel, there was an essentially unsuccessful unilateral attempt by the US to force China to reform its economy.

Not surprisingly, then, the failure of President Trump to win re-election induced sighs of relief in the European halls of power. During the past two months, many proposals have been advanced to revitalise transatlantic cooperation on a host of climate change, economic, geopolitical and trade-related matters. Even before the Biden administration took office, the European Commission (2020) laid out its wares in a formal Communication on potential future transatlantic cooperation. In addition, President Trump's defeat has been accompanied by a volley of commentary on the prospects for greater cooperation across the Atlantic (the more insightful of which in the opinion of this author are Demertzis, 2020; Freudlsperger et al., 2020; Odgaard, 2020; Köhler-Suzuki, 2020; van Daniels et al., 2020; and Williams, 2020).

In such commentary, mention is often made of the political constraints and imperatives in the United States and in European nations (including the growing desire of "strategic autonomy" on the part of the latter), of the continuing fallout from the coronavirus pandemic and the near-term challenges created for policymakers, of the relative success of state-driven forms of capitalism as compared to liberal democratic alternatives, of the longer term global challenges such as climate change, of the malaise at certain international organisations (such as the World Trade Organization) and of the growing economic, military and technological rivalry between the world's leading powers.[1] The context, then, of future transatlantic cooperation is multi-faceted to say the least.

The focus of this paper is on one recurring and prominent feature of recent proposals for strengthened transatlantic cooperation – namely to counter state-driven forms of capitalism. This is taken here to refer principally to the Chinese economic model but arguably includes Russian, South African and other emerging market variants. Claims made in such proposals that transatlantic cooperation should tackle the Chinese Dragon and has the means to do so are scrutinised and found wanting. This is not the end of the matter – after all, the Biden administration has

**Simon J. Evenett**, University of St. Gallen, Switzerland; and Centre for Economic Policy Research, London, UK.

1 Interestingly, very few commentators discuss the implications of China's new economic policy of "dual circulation" for transatlantic cooperation. This is surprising given the apparent desire of Chinese policymakers to rebalance economic growth towards domestic demand and to reduce sourcing from ("dependence" upon) suppliers abroad. Both would have potential implications for how much Western firms can sell to China and their market entry strategies as well as for Chinese support to its own exporters and the adverse cross-border spillovers that such support may engender.

yet to take office. But the following discussion highlights the prerequisites for translating stated intentions into a coherent international economic strategy.

Specifically, in this respect, it is worth asking whether any transatlantic counter to state-driven capitalism can convincingly answer the following questions:[2]

- What is the *purpose* of the strategic counter? What evidence is there that the proposed strategy has identified tangible, first-order[3] problems faced by European and North American nations? First-order for whom? Is the objective to induce the reform of state-driven capitalism, to protect against the adverse effects of such capitalism, or both?

- What *guiding policy* has been developed as to how to attain this purpose? What capabilities are required? Critically, what is the Theory of Change that relates those capabilities and the coherent policy to the desired outcomes? Is that Theory cogent? Does the future application of that Theory have convincing, relevant precedents?

- What *coherent actions* would follow from the adoption of the guiding policy? By whom and with whom? Over what time frame? Will the actions that follow from the guiding policy compromise other cherished policy objectives? If cooperation between states is to be codified, what instruments will be deployed and how will compliance be induced and verified?

Asking these questions is essential if policymakers are to avoid going down blind alleys, influenced perhaps by certain corporate interest groups to pursue initiatives that are lucrative for them but ultimately of second or third order. It is also necessary to avoid the pursuit of initiatives that have little or no chance of influencing policymaking in Beijing or indeed any other government whose current policies are deemed objectionable.[4]

2 Those with a business school training will recognise that the three following questions are adaptions of Richard Rumelt's (2011) persuasive approach to the formulation and evaluation of corporate strategy. An important adaption here is the additional requirement that there be a compelling Theory of Change; in this case, for example, a theory of which factors increase the likelihood that the government in Beijing modifies or reforms its economic model.

3 Here it is useful to ask if the problem or problems can be scaled? This shifts deliberation from specific examples (which can be hammed up into war stories) to specific, ideally quantitative, evidence.

4 Although framed in this paragraph in terms of influential corporate interests, similar concerns can arise in conjunction with the proposals of non-government organisations, including civil society groups and trade unions.

It is also worth noting that the first terms of the von der Leyen Commission and the Biden administration both conclude within two months of one another. Given the time it takes for a US Administration's senior officials to get confirmed and master their briefs, essentially officials on both sides of the Atlantic have approximately three years to design and execute a new approach to transatlantic cooperation before the next US presidential election is in full swing. This is not a long period of time and it ought to induce harder thinking than has been witnessed to date. Officials on both sides of the Atlantic need to choose their battles wisely. Consequently, the evidentiary bar that a proposed initiative must meet should be set high.

To provide grist for the mill, the next section provides summaries of two high-profile, very recent proposals for enhanced transatlantic cooperation. Both proposals give pride of place to countering authoritarian regimes and their state-driven economic models. Those proposals are then assessed in terms of the answers implicitly provided to the three questions listed above and implications are drawn concerning the prospects of transatlantic trade cooperation taming the Chinese Dragon.

## Hot to trot: Two high-profile proposals to revitalise transatlantic cooperation

More ink has likely been spilt on transatlantic relations during the two months since the US presidential election than over the previous five years. To provide a sense of the substance and apparent logic being advanced, two high-profile recent initiatives are summarised here. Readers are encouraged to bear in mind the three questions raised in the section above when reflecting on the excerpts that follow.

### A new EU-US agenda for global change

On 2 December 2020, the European Commission published a Communication titled *A new EU-US agenda for global change*. While recognising the importance of transatlantic cooperation in the past, not least in rebuilding Europe after World War II, the Communication contends that the future cooperation must be grounded in today's circumstances: "The US and the EU have changed, as have power dynamics and geopolitical and technological realities" (European Commission, 2020, 1). In terms of the status quo, the European Commission (2020,1) contends: "Today, our combined global power and influence remains unrivalled".

Moving closer to a general statement of intent, the European Commission (2020) puts the need for greater cooperation in key areas of policy in the context of the rise of non-democratic powers:

> This combined power and influence is indispensable to anchor global cooperation in the 21st century – whether it be on health, security, climate, trade and technology, or on the multilateral rules-based order. Our joint commitment is essential in a world where authoritarian powers seek to subvert democracies, aggressive actors try to destabilise regions and institutions, and closed economies exploit the openness our own societies depend on. (1)

The Communication then turns to the specifics of cooperation on health, climate change, technology and the digital economy, trade, democracy and foreign and security policy. Interestingly, the discussion on health policy focuses exclusively on what the EU and US can do together or on unilateral initiatives the US could take. No mention is made of inducing changes in policy by non-democratic nations. The same is true for climate change (and the related discussion on biodiversity).

On technology and trade matters, the European Commission (2020) acknowledges: "While we are still the most influential regulators, both the EU and the US face increasing standard competition from third country actors. Where both sides agree, the world usually follows" (7). This statement is a fragment of a Theory of Change. This followed a discussion of the threats posed by non-democratic nations to digital supply chain security, cybersecurity and the regulation of advanced digital technologies more generally. Little is said about how to tackle those threats in practice.

While reference is made to unilateralism in trade policy and the desire to promote transatlantic trade and investment flows, the attention given to resolving long-standing trade frictions is cursory.

With respect to promoting democracy, human rights, and labour rights, the European Commission sees potential for greater transatlantic cooperation. The Commission is willing to participate in a Summit for Democracy (thought to be a priority of the Biden administration) and to join others in making "further commitments on fighting corruption, authoritarianism and human rights abuses around the world" (European Commission, 2020, 8). Notice there is no claim that those commitments would be taken on by other nations.

In the ensuing discussion on making the world safe, prosperous and more democratic, the Communication singles out China:

> For the EU, China is a negotiating partner for cooperation, an economic competitor, and a systemic rival… As open democratic societies and market economies, the EU and the US agree on the strategic challenge presented by China's growing international assertiveness, even if we do not always agree on the best way to address this. (European Commission, 2020, 8)

This vague statement of objectives is a good example of those that pervade the Communication. Is the goal to protect EU and US interests from Chinese "assertiveness"? To protect the rest of the world from such assertiveness as well? To change Chinese behaviour, perhaps moderating Beijing's apparent assertiveness? Or all of the above? These goals are not the same. Nor necessarily are the guiding policies and coherent actions that would follow from a strategy to pursue each of them.

Overall, when compared to the documents issued by the European Commission at the time the Transatlantic Trade and Investment Partnership was launched in 2013, the European Commission's position has shifted markedly. Geopolitical, climate change and digital technology initiatives take centre stage now, essentially pushing the resolution of long-standing transatlantic trade and investment disputes to the side. As will become evident, another recent high-profile set of proposals for transatlantic cooperation was not prepared to go that far.

### Stronger Together: A Strategy to Revitalize Transatlantic Power

Harvard University's Belfer Center joined forces with the German Council on Foreign Relations (DGAP) to form a Transatlantic Strategy Group comprising of 16 established experts and former diplomats. In December 2020 this group published a report titled *Stronger Together: A Strategy to Revitalize Transatlantic Power*. Their headline recommendation is that "[t]he United States, Europe and Canada must work together toward one ambition in 2021—to renew, revitalise, and retool for the decade ahead the most powerful democratic community in modern history" (Harvard-DGAP, 2020, 2).

Looking beyond "years of mistrust, recrimination, and division," nations on both sides of the (north) Atlantic must cooperate because "the world needs a more powerful and purposeful transatlantic alliance to drive a new global agenda" (Harvard-DGAP, 2020, 2). In addition to tackling climate change, the following end is given prominence:

> We must also harness our joint power to deter a more confident and aggressive China and a cynical and disruptive Russia. They have exploited transatlantic ten-

> sions for too long. Together we must oppose their illiberal agendas in Europe, Africa, the Indo-Pacific, and around the world. (Harvard-DGAP, 2020, 3)

A wide range of objectives is then laid out: rebuilding trust, revitalising democracies at home, a joint strategy to tackle global challenges and to "defend liberalism", and transformation of "our political, military, technological and economic capacity to be the most effective force for freedom and rules-based order in a challenging world" (Harvard-DGAP, 2020, 9).

Action plans for eight areas of policy were then advanced, of particular interest here are those relating to Economics and Trade and to China. With respect to the former, although the action plan is motivated by various 'challenges' posed by China's rise, the preponderance of the Action Plan relates to steps the EU and the United States can take to reduce trade frictions between themselves. Of the 17 specific trade-related steps articulated on pages 19 and 20 of this report, only two specifically refer to China and another two items (subsidies and supply chain resilience) relate to matters said to arise from Chinese policy. Moreover, earlier in the Action Plan it was recognised that to date the EU and the US have had different interests and approaches to key trade policy matters, including dealing with China's growing clout.

The latter point is reinforced in the Action Plan on China where it is argued that European Union member states often see Chinese relations in terms of economic or trade ties, whereas the US regards China as a threat to its primacy. It is argued that these differences must be overcome now that China's assertiveness materially affects the European Union and the United States, in the following ways:

> The transatlantic community is not immune to Beijing's assertiveness. The U.S. and Europe share concerns over Chinese forced technology transfers; trade subsidies; issues over reciprocity and market access for U.S. and European companies; surveillance technologies; and political influence associated with its economic investments. There is also increasing support in the U.S. and across Europe for pushing back against Chinese human rights abuses and developing global standards in emerging technologies as China advances its own digital authoritarian model. (Harvard-DGAP, 2020, 36)

Having described the evolving hardening of positions within Europe and the United States, the Harvard-DGAP report articulates the following Theory of Change:

> Only together can the U.S. and Europe build the leverage necessary in trade, technology and multilateral engagement to hold China to a set of standards that protects democratic societies and contributes to global stability. United they can rally other nations around these objectives. (Harvard-DGAP, 2020, 39)

The following trade and investment-related recommendations are advanced in the Action Plan for China:

- Bring joint cases to the WTO that "prioritize Chinese intellectual property theft, uncompetitive trade practices and cybertheft" (Harvard-DGAP, 2020, 39).

- Forming "a U.S.–EU working group on WTO reform could serve as an initial step in assessing how best to address Chinese uncompetitive trade practices" (Harvard-DGAP, 2020, 39).

- Strengthen and align polices that screen foreign direct investments, in particular those relating to critical infrastructures.

- Increased intelligence sharing "on cyber espionage, forced technology transfers and IP theft" (Harvard-DGAP, 2020, 40).

- "Technology transfers to China through collaboration in research institutions and universities must be more closely scrutinized" (Harvard-DGAP, 2020, 40), with particular emphasis on collaboration with European universities.

- Develop an alternative to China's Belt and Road Initiative by providing "transparent finance and infrastructure to developing countries that are growing increasingly indebted to Chinese loans and infrastructure projects" (Harvard-DGAP, 2020, 41).

- Greater collaboration to "prevent sensitive technology transfers to Chinese companies and military actors involved in surveillance. Collaboration should also focus on developing joint standards to evaluate relevant transactions" (Harvard-DGAP, 2020, 41-42).

### Where their paths cross

There are clear parallels to some of the framing of both proposals: confronting authoritarian regimes and state capitalism being perhaps the most important examples.[5] Not surprisingly, then, China has pride of place in

5 However, the two initiatives are not completely aligned (and there is no reason why they must be). The Harvard-DGAP report puts greater store on resolving existing transatlantic trade frictions. It is as if the European Commission's Communication has been drafted to signal "let's not let old sores get in the way of future cooperation."

both proposals. The scope of both – encompassing climate change, geopolitical matters, human rights, etc. – are similar and far broader than that envisaged when the Transatlantic Trade and Investment Partnership was being negotiated. This raises the important question as to whether traditional trade policy initiatives will be subordinate to other policy imperatives, possibly being demoted, bargained away, taken up later or even dropped? Or whether geopolitical and other considerations are used to overcome some of the longstanding impediments to classic forms of transatlantic trade cooperation?

Having laid out these two proposals, that are representative of many of those which have called for closer transatlantic cooperation in recent months, the discussion now turns to whether they constitute fully fledged strategies in the sense articulated in the last section.

### Critical assessment: Falling short as strategy

To what extent do the European Commission's Communication and the Harvard-DGAP study proposals constitute well-formulated strategies? Some may object that it is too much to expect that these documents lay out in full detail the purpose, guiding policy and cogent actions associated with their proposals for future transatlantic cooperation. Even if this were a fair comment, then should these proposals be taken forward, those details need to be worked through and the following discussion may be useful at that stage.

To sharpen ideas, the following discussion focuses on the multiple references in these two documents that amount to, at a minimum, countering Chinese influence and possibly seeking to change Chinese government behaviour. Although taming China is in vogue, many of the following remarks apply with equal force to attempts to influence government decision-making in, for example, Ankara, Moscow and New Delhi.

With respect to the purpose of taming the Chinese Dragon, while Chinese actions are alluded to, the lack of specificity concerning Chinese ends, means and consequences for other nations is alarming. Prioritisation of Chinese threats (if that is what they are) is going to be very difficult under these circumstances.

For example, if excess capacity in Chinese manufacturing industry is a problem, how big a problem is it? Are the problems bigger in some manufacturing sectors than others? In assessing how big a problem there is in any one sector, what evidence is there that increased excess capacity results in increased Chinese exports, in increased imports into Europe and into the United States, and into greater tangible harm to import-competing firms and their employees? (Bear in mind that imports may rise because demand in the importing nations for the products in question has risen – so higher imports from China do not automatically signify larger excess capacity in Chinese manufacturing).

In short, a lot of work needs to be done to determine which threats, if any, from China are really first order. In the absence of this groundwork, there is a risk that national policymaking and transatlantic cooperation will be hijacked by influential corporate interests that exaggerate the importance of well-chosen examples.[6] Evidence, not war stories and the fear they engender, should underpin any new phase in transatlantic cooperation.

With respect to guiding policy, it is difficult to discern any from either document advocating enhanced transatlantic cooperation. To draw a historical parallel, if taming the Chinese Dragon is really the goal, then what is needed is the contemporary equivalent of George Kennan's 1946 telegram to the US State Department that outlined a strategy to "contain" the Soviet Union. In places, there are statements that might be part of a plausible candidate Theory of Change – an example being the quote from page 39 in the Harvard-DGAP study reproduced above. Such examples should be scrutinised carefully.

For instance, suppose the EU and US agree on a common approach to a particular regulatory problem. What incentives are there (or could be created) to induce other nations to "rally round" and sign up to such an initiative? How many nations can plausibly be expected to do so and how much economic activity would they cover? Which nations are likely to refuse to join – and do they constitute a critical mass of their own? Particular focus should be given as to whether China would see it in its interests to sign up.

More generally, it is worth differentiating between the Theories of Change purporting to induce Beijing to reform elements of its foreign economic policy and to reform its domestic economic, social and political policies. Depending on the terms, a multi-party deal to reform certain aspects of international development finance might be more acceptable to Beijing than agreeing to reforms to establish independent trade unions.

6 The author's suspicions on this score arose after conducting a detailed examination of claims made about the adverse consequences of Chinese excess capacity in the steel sector (Evenett and Fritz, 2018).

Evidently, taking account of the factors that drive Chinese policymaking is critical. Yet in the preparation for this paper, which involved reading hundreds of pages of text on proposals and commentary on the future of transatlantic relations published over the past two months, only *one* document made reference to *one* of the well-known books on Chinese economic statecraft.[7] If taming the Chinese Dragon is the goal, then surely this requires some understanding of policymaking in Beijing? In the absence of such understanding, there is little in current proposals to keep defenders of the status quo in Beijing awake at night.

There is another troubling feature of the two proposals described here and it relates to the "leverage" of the European Union and the United States. Repeatedly, readers are informed that together the transatlantic nations' "global power and influence remains unrivalled", "indispensable" (European Commission, 2020, 1), etc. Here it is worth differentiating between positive and negative liberty, along the lines advanced by Isaiah Berlin (1958), but reinterpreted here for governments rather than individuals.

Together, the EU and the United States may be able to block more initiatives that are not to their liking. However, such negative liberty does not imply that the transatlantic economic powers have positive liberty – that is, the capacity to persuade other governments to sign up to their initiatives. Clearly, there is a link between the capability and likelihood of gaining acceptance of one's own proposals for global norms (positive liberty in this sense) and the adopted Theory of Change, as the latter relates to the willingness of other governments to accept and comply with those norms.

Indeed, although both documents reviewed here were thin on specifics, they tended to be thinner on precisely what the European Union and the United States could plausibly accomplish when persuading third parties to follow their lead and how that might come to pass. If this relative lack of detail reflects doubts after decades of limited multilateral trade cooperation about the true degree of leverage that these two economic "powers" can muster in commercial policy matters in the 21st century, then greater emphasis might be put on accommodating different forms of capitalism in the world economy and on building defences against the negative spillovers created by rival capitalisms.

7 It is noteworthy that for a report that put so much weight on countering China, the Harvard-DGAP group did not include a single recognised expert on Chinese policymaking.

Finally, important questions arise concerning the coherence of the transatlantic and other international initiatives that might be undertaken in the years ahead.[8] If, as both EU and Biden administration officials have stated, China's cooperation is needed to tackle climate change, then how much can transatlantic cooperation hope to accomplish in inducing China to change policies in areas where it is reluctant to do so? To what degree can initiatives with Beijing and those targeting Beijing be compartmentalised? If issue-linkage develops and progress on climate change, say, requires going slow on tackling subsidies, intellectual property theft or cybersecurity, then is such a trade-off acceptable in European capitals and Washington, DC? And can the latter two remain aligned when such trade-offs arise?

For all of these reasons, the leading proposals for enhanced transatlantic cooperation fall short. In the form published, they do not rise to the standard of a well-thought through strategy. That is not to imply that no compelling strategy is possible, rather, that readers should curb their enthusiasm until such a blueprint is presented by officials. "The level of ambition on both sides of the Atlantic is currently sky high", as Williams (2020) put recently, but such ambition has yet to translate into a serious strategy – let alone one that could tame the Chinese Dragon.

## Concluding remarks: Girding their loins but then what?

After four of the worst years for transatlantic relations since the World War II, it is only natural that many policymakers and analysts want to turn the page. Moreover, that the world has moved on implies that the next chapter of transatlantic cooperation, if there is to be one, will probably look different from the past. This is just as well for, at least as far as trade policy cooperation is concerned, arguably little of first-order importance has been accomplished since European nations and the United States joined forces to create the World Trade Organization a generation ago.

Still, being keen to cooperate and establishing a sound basis upon which to cooperate are two very different matters. As the past two months have demonstrated, the appetite is there but evidence of hard thinking is scarce.

8 Although the observations made here relate to the coherence among international initiatives that the European Union and the United States might undertake together, of course, the coherence of each party's international initiatives with its domestic policy imperatives is another pertinent consideration. For example, Mr. Biden is on record stating that no new trade agreements will be negotiated while the United States takes steps to restore its domestic competitiveness.

Perhaps, officials at the European Commission and in the Biden Transition Team have done their homework and are keeping it to themselves; however, given the ways in which they are framing their arguments, this is doubtful.

The desire of many policymakers to tame the Chinese Dragon is apparent. But what is particularly disappointing is the little, if any, reflection on how to induce the government in Beijing to change course, whether with respect to domestic policy or foreign economic policy. If this is a consequence of doubts about the leverage of European and American policy then, whether intended or not, attention is likely to shift to how tensions can be better managed with Beijing or, worse, to whether further decoupling of economic and governance processes is necessary. Hardliners, who have had the upper hand in the past decade, are likely to favour the latter outcome.

This is not an argument in favour of decoupling. Rather it is an argument that, unless a cogent strategy is formulated with some sense of a plausible alternative endgame, then there is a risk of sleepwalking into a scenario of extensive decoupling. Such decoupling is a recipe for the fragmentation of the global economic and governance processes, with its attendant losses in terms of specialisation and productivity, collaboration on innovation, and opportunities for dialogue and engagement. If fragmentation is the ultimate, unintended by-product from attempts to revitalise transatlantic cooperation over the next four years, then historians may look back at this era and wonder if the outcome of the November 2020 US presidential election really mattered.
